# Predictive modelling approaches for childhood diarrhoeal disease: a systematic review (2000–2025)

**DOI:** 10.1186/s40249-026-01508-8

**Published:** 2026-11-03

**Authors:** Sunyun Qi, S M Nuruzzaman Nobel, Amutha Ramadas, Hua Gu, Kia Fatt Quek

**Affiliations:** 1 Jeffrey Cheah School of Medicine and Health Sciences, Monash University Malaysia, Jalan Lagoon Selatan, Bandar Sunway, 47500 Selangor Darul Ehsan Malaysia; 2 Department of Electrical and Robotics Engineering, School of Engineering, Monash University Malaysia, Jalan Lagoon Selatan, Bandar Sunway, 47500 Selangor Darul Ehsan Malaysia; 3 Zhejiang Evaluation Centre for Medical Service and Administration, Hangzhou, 310005 China

**Keywords:** Childhood diarrhoea, Prediction model, Machine learning, Systematic review

## Abstract

**Background:**

Childhood diarrhoeal disease remains a leading cause of child mortality worldwide. Although predictive models have been increasingly proposed for early warning, their methodological quality and validation have not been systematically appraised using formal risk of bias frameworks. This systematic review aimed to identify, describe, and critically appraise individual-level prediction models and population-level forecasting studies for childhood diarrhoeal outcomes, focusing on model development, validation, reporting quality, and operational readiness.

**Methods:**

Following PRISMA 2020 guidelines (PROSPERO: CRD420251232269), we searched seven databases and grey literature sources from January 2000 to September 18, 2025. We included studies that developed or validated multivariable prediction models for diarrhoeal outcomes in children, including statistical, machine learning, and hybrid approaches. Individual-level models were assessed using the Prediction Model Risk of Bias Assessment Tool (PROBAST), and population-level studies were appraised qualitatively for methodological credibility, with emphasis on validation.

**Results:**

From the 20,662 records screened, 38 studies met the inclusion criteria. Publications accelerated after 2020, with 78.9% of included studies published in this period. The evidence base comprised 25 single-country studies and 13 multi-country or global analyses. Statistical models were most commonly used, as reported in 18 studies, while ensemble or hybrid approaches such as hybrid statistical–machine learning models were applied in 11 studies, and machine learning methods in eight studies. Predictor selection varied widely, with socio-demographic and household factors included in 23 studies and environmental or climatic variables in 18. Among the 15 individual-level prediction models assessed using PROBAST, five showed high overall risk of bias. Of the 19 population-level forecasting studies appraised qualitatively, only three undertook rigorous out-of-sample temporal validation. The remaining four studies were classified as other modelling categories and were summarised descriptively.

**Conclusions:**

Research on childhood diarrhoeal prediction has expanded rapidly across individual-level, population-level, and related classification models. Although some studies reported stronger validation practices, these remained uncommon, leaving evidence for transportability and implementation readiness limited for most models. Future progress will depend on closer alignment between modelling objective, validation strategy, appraisal framework, and transparent reporting.

**Graphical Abstract:**

**Figure:**
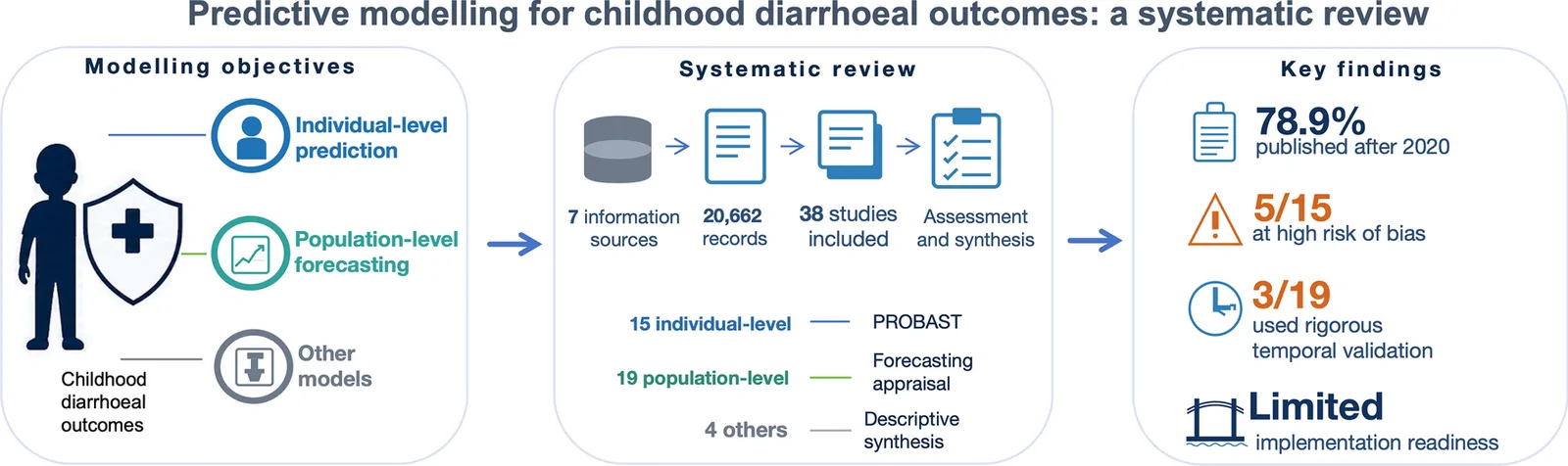

**Supplementary Information:**

The online version contains supplementary material available at https://doi.org/10.1186/s40249-026-01508-8.

## Background

Childhood diarrhoeal disease remains a major global health challenge among children under five. In 2023, diarrhoeal diseases caused an estimated 317,000 deaths (95% UI: 213,000–462,000) among children younger than five years worldwide, with the burden concentrated in low- and middle-income countries where inadequate water and sanitation, undernutrition, and limited access to timely care increase both incidence and severity [1, 2]; recurrent episodes may further contribute to growth faltering, impaired immune function, and poorer cognitive development [3]. Despite effective interventions, preventable morbidity and mortality persist [4], while routine surveillance is often retrospective and operationally constrained, limiting anticipatory action [5]. Prediction models and early warning approaches may complement surveillance by forecasting near-term burden, identifying high-risk populations or locations, and supporting targeted allocation of scarce resources [6]. This is particularly relevant because childhood diarrhoeal risk varies with climatic and environmental exposures, household and socioeconomic determinants, and depending on modelling objective, child-level clinical features [7–11]; in the context of climate variability and change, transportable tools may also support preparedness and response planning in resource-limited settings [12–16].

Predictive modelling for childhood diarrhoeal disease has expanded rapidly in scope and methodological diversity. Studies have utilized statistical time-series and regression models alongside machine learning, ensemble, and hybrid approaches [6, 17, 18], drawing on data sources that now extend beyond routine surveillance to include meteorological observations, satellite products, population-based surveys, electronic health records, and other environmental or digital streams [19, 20]. However, the evidence base remains fragmented, with substantial heterogeneity in outcome definitions, predictor selection, modelling paradigms, validation strategies, and performance reporting, limiting comparability and complicating translation into operational tools [21].

A first major gap is confusion in model classification. Although these approaches are often grouped together under the broad label of ‘predictive models’, they differ fundamentally in their unit of prediction, intended use, and required validation strategy. Individual-level clinical prediction models estimate risk or classify outcomes for a child, whereas population-level forecasting models predict aggregated counts, incidence, outbreaks, or temporal trends. Etiological and pathogen-oriented classification models represent a further distinct category. When these modelling objectives are not clearly differentiated, inappropriate validation standards may be applied and model performance may be interpreted misleadingly [22, 23]. In particular, the field lacks a consistent distinction between validation expectations for individual-level prediction and those for population-level forecasting.

A second major gap relates to the lack of consistent validation standards across modelling objectives. Many studies rely primarily on internal procedures such as split-sample evaluation or cross-validation, which may yield optimistic performance estimates and provide limited evidence of transportability to new settings, populations, or time periods [24]. For individual-level prediction models, external validation and calibration are especially important because good discrimination alone does not guarantee clinically reliable risk estimates [25]. For population-level forecasting studies, credibility depends on true out-of-sample temporal evaluation rather than retrospective in-sample fit. Yet across this literature, validation practices remain inconsistent, with greater emphasis often placed on internal fit or algorithmic novelty than on external validation, calibration, and real-world robustness.

A third major gap is the absence of an appraisal framework that aligns quality assessment with the intended modelling objective. Transparent reporting and systematic critical appraisal are essential to determine whether published models are credible and implementable. The Prediction Model Risk of Bias Assessment Tool (PROBAST) provides a structured approach to evaluate bias arising from participant selection, predictor and outcome measurement, and analytical methods in individual-level prediction studies [26, 27]. However, PROBAST was not designed for population-level forecasting, ecological time-series analyses, or long-term burden projections, which require complementary epidemiological appraisal focused on issues such as temporal validation, seasonality, non-stationarity, and forecasting credibility [28]. At the same time, the translation of predictive models into practice is hindered by a broader reproducibility crisis, in which increasingly complex algorithms often lack sufficient transparency for independent verification [29]. Together, these issues highlight the need for a review framework that both distinguishes modelling objectives and applies quality appraisal methods appropriate to each type of study.

Despite these challenges, childhood diarrhoeal disease represents an important context for methodological evaluation because its occurrence and severity are influenced by interacting climatic, environmental, socioeconomic, and clinical determinants. This complexity generates diverse prediction tasks spanning individual-level risk prediction, population-level forecasting, and pathogen-oriented classification. Reliable prediction models may support earlier outbreak detection, targeted public health interventions, and more efficient allocation of scarce healthcare resources. Strengthening predictive capability for childhood diarrhoeal disease may therefore support progress towards Sustainable Development Goal (SDG) 3.2, which aims to end preventable deaths of newborns and children younger than five years by 2030 [30].

To address these gaps, we conducted a systematic review to identify, describe, and critically appraise individual-level prediction models and population-level forecasting studies for childhood diarrhoeal outcomes, with particular attention to model development, validation, reporting quality, and operational readiness. Specifically, we differentiated between individual-level prediction, population-level forecasting, and etiological classification tasks. We then applied PROBAST to eligible individual-level multivariable prediction models and used a complementary epidemiologically informed appraisal for population-level forecasting studies.

To structure our review, we developed a conceptual framework that distinguishes between key modelling objectives. This framework, summarised in Table 1, guided data extraction and interpretation by linking each modelling objective to its critical validation requirements and public health goals. A more detailed version, including typical modelling approaches and implementation contexts, is provided in Additional file 2.

**Table 1:** Conceptual framework linking modelling objectives to validation requirements and public health goals

Modelling category	Critical validation standard	Public health goal
Individual-level risk prediction (diagnosis, prognosis, classification)	External validation: performance in new populations; calibration: accuracy of risk probabilities	Clinical triage; targeted treatment; etiological diagnosis
Population-level forecasting (early warning, time-series)	Out-of-sample testing: forecasting future time points; horizon accuracy at specific lead times	Outbreak detection; resource stockpiling (short-term)
Long-term projection (burden estimation, scenario analysis)	Sensitivity analysis: robustness to assumptions; trend consistency with historical patterns	Strategic planning; climate adaptation; vaccine policy

This conceptual framework distinguishes modelling objectives rather than final appraisal strata

## Methods

### Search strategy

The review protocol was registered in the International Prospective Register of Systematic Reviews (PROSPERO; CRD420251232269) on 16 November 2025 [31]. Registration was completed after the literature search had commenced. No major amendments were made to the protocol after study screening commenced. This systematic review was conducted and reported in accordance with the Preferred Reporting Items for Systematic Reviews and Meta-Analyses (PRISMA) 2020 statement [32], and the development and reporting of electronic search strategies followed PRISMA-S [33].

A comprehensive search was conducted on 18 September 2025 in PubMed/MEDLINE, Scopus, Web of Science Core Collection, Embase (via Ovid), and Global Health (via Ovid), including English-language records published or available online between 1 January 2000 and 18 September 2025. To identify grey literature, we additionally searched Google Scholar and ProQuest Dissertations & Theses Global. For Google Scholar, we screened the first 100 records sorted by relevance using simplified keyword combinations aligned with the database search concepts. For ProQuest Dissertations & Theses Global, we applied analogous keyword blocks and screened all records returned after applying date and language limits. Reference lists of included studies and relevant reviews were also checked for additional eligible records.

Search strategies were tailored to each database and combined controlled vocabulary terms, including MeSH and Emtree, with free-text terms across three domains: paediatric populations, diarrhoeal diseases and enteric infections, and prediction, modelling, and forecasting approaches. The full reproducible search strategies for all sources are provided in the Additional file 3. All records were exported to Zotero (version 7.0.32; Corporation for Digital Scholarship, Vienna, VA, USA) [34] for initial management and subsequently imported into Covidence (Veritas Health Innovation, Melbourne, VIC, Australia) [35] for deduplication and screening. To minimise the risk of missing relevant studies, grey literature sources and reference lists of included studies and relevant reviews were also screened. Formal statistical assessment of reporting bias was not undertaken because substantial heterogeneity in study designs, modelling objectives, and performance metrics precluded meaningful quantitative comparison [36].

### Eligibility criteria

Studies were eligible if they developed, validated, or applied a quantitative modelling framework intended for prediction, prognostication, classification, early warning, or forecasting of diarrhoeal disease outcomes, including both human-population models and pathogen genomic sequences. Only studies published in English were considered. We focused on children under five years of age. Studies enrolling mixed-age populations were included only if under-five results were clearly reported or could be extracted. For studies involving broader populations or non-obvious outcome definitions, study-specific eligibility justifications are provided in Additional file 4 to facilitate verification of eligibility decisions.

Eligible outcomes included diarrhoeal incidence, prevalence, case counts, mortality, hospitalisation, pathogen-specific detection or etiological classification, outbreak occurrence, hotspot identification, and individual- or population-level risk prediction or forecasting. We included statistical models, machine learning, deep learning, and hybrid approaches, and required at least one quantitative performance evaluation appropriate to the modelling task, such as discrimination measures for classification models or error-based metrics for forecasting models. Genomic and pathogen classification studies were included when they developed or evaluated computational models intended to predict, classify, or identify diarrhoeal pathogens or diarrhoeal aetiology using genomic, molecular, or sequence-based data. Studies focused solely on genomic characterisation, phylogenetic description, molecular epidemiology, or evolutionary analysis without a predictive or classification modelling component were excluded.

We excluded descriptive epidemiological studies without model development or evaluation, and studies conducted exclusively on animals or in laboratory settings without population-relevant prediction. We also excluded publications lacking sufficient methodological detail to permit understanding or appraisal of the modelling approach. Specifically, a study was excluded if it did not adequately report one or more of the following core elements: the modelling method or algorithm, predictor variables or input features, outcome definition, data source or study population, validation or evaluation procedure, and at least one appropriate model performance measure. We also excluded review articles, editorials, commentaries, study protocols, case reports, and conference abstracts without a corresponding full-length paper, as well as studies focused exclusively on adults with no extractable under-five results.

### Study selection and data extraction

After deduplication, two reviewers (SQ and SMNN) independently screened titles and abstracts in Covidence against the eligibility criteria. The deduplication process followed standard systematic review principles to ensure unique record identification [37]. Full-text articles were obtained for all records considered potentially eligible by either reviewer and were assessed independently by the same reviewers. Disagreements were resolved through discussion, with a third reviewer (KFQ) consulted to adjudicate unresolved conflicts.

Data were extracted using a standardized form developed based on the CHARMS checklist [38]. The extraction form was pilot-tested on a random subset of five included studies and refined iteratively to ensure consistency. One reviewer (SQ) performed the primary data extraction, which was independently verified for accuracy and completeness by a second reviewer (SMNN). Extracted items covered study and population characteristics, dataset and outcome definitions, predictor variables, modelling approach, validation methods, and reported performance measures. Where reported, we also extracted information on the intended decision context or operational use of each model.

### Risk of bias assessment and quality appraisal

For studies developing or validating individual-level multivariable prediction models, two reviewers (SQ and SMNN) independently assessed methodological quality using PROBAST [39]. PROBAST evaluates risk of bias across four domains: Participants, Predictors, Outcome, and Analysis. Disagreements were resolved through discussion or, when necessary, by adjudication from a third reviewer (KFQ). Overall risk of bias was judged according to official PROBAST guidance. A study was rated as low overall risk only when all four domains were judged as low risk; it was rated as high overall risk if one or more domains were judged as high risk; and unclear risk was retained when reporting was insufficient to support a confident judgement. All data management and visualisations were performed using the R statistical computing environment (version 4.5.2; R Foundation for Statistical Computing, Vienna, Austria) [40] and the RStudio IDE (version 2026.01.0; Posit Software, PBC, Boston, MA, USA) [41]. Additionally, functions from the following packages were used for data preparation, visualization and analysis: tidyverse [42], dplyr [43], tidyr [44], scales [45], and ggplot2 [46].

To align quality appraisal with modelling objectives, included studies were assigned to three appraisal strata: individual-level prediction studies, population-level forecasting studies, and other modelling categories. Additional file 9 provides a study-level summary table showing how each included study was classified by modelling objective and appraisal stratum, and which appraisal method was applied. Individual-level prediction studies were assessed using PROBAST. Population-level forecasting studies were appraised qualitatively using an epidemiologically informed framework, whereas other modelling categories, including pathogen/genomic classification and unsupervised clustering analyses, were summarised descriptively because they fell outside the intended scope of both PROBAST and the forecasting appraisal framework.

Because PROBAST is specifically designed to assess risk of bias and applicability in individual-level prediction models [39], studies whose primary objective was not individual-level prediction were not assessed using PROBAST. Population-level forecasting studies were appraised using four predefined criteria informed by the EPIFORGE guidelines [47]: (i) data completeness and temporal resolution; (ii) handling of seasonality and non-stationarity [48]; (iii) use of true out-of-sample temporal validation; and (iv) appropriateness of forecasting performance metrics [49]. To improve transparency and reproducibility, study-level judgements for these criteria are provided in Additional file 10. This table operationalises the predefined appraisal criteria only and does not constitute an additional scoring system, risk-of-bias framework, or overall rating.

Genomic and pathogen-classification studies were summarised descriptively and were not subjected to formal risk-of-bias assessment because no established appraisal framework was identified that was directly applicable to this category of modelling study.

For the Analysis domain, official PROBAST signalling questions were applied [39]. Internal validation procedures, including cross-validation, bootstrap resampling, and split-sample validation, were considered acceptable for model-development studies [25]. Studies were not judged at high risk in the Analysis domain solely because they lacked external validation or calibration assessment.

### Evidence synthesis and analysis

Given substantial heterogeneity in populations, outcomes, prediction horizons, predictors, modelling approaches, and reported performance metrics, quantitative meta-analysis was not feasible [36]. Findings were therefore synthesised narratively using structured grouping by modelling objective, study setting, data source, predictor domain, validation strategy, and reported performance [50, 51].

The synthesis explicitly distinguished between individual-level prediction models, population-level forecasting studies, and other modelling categories. PROBAST results were summarised for eligible individual-level models, whereas population-level forecasting studies were summarised using the predefined qualitative appraisal criteria. Risk-of-bias findings, validation practices, and methodological credibility were integrated into the interpretation of results. We did not apply a separate certainty-of-evidence framework.

## Results

### Study selection

**Fig. 1:**
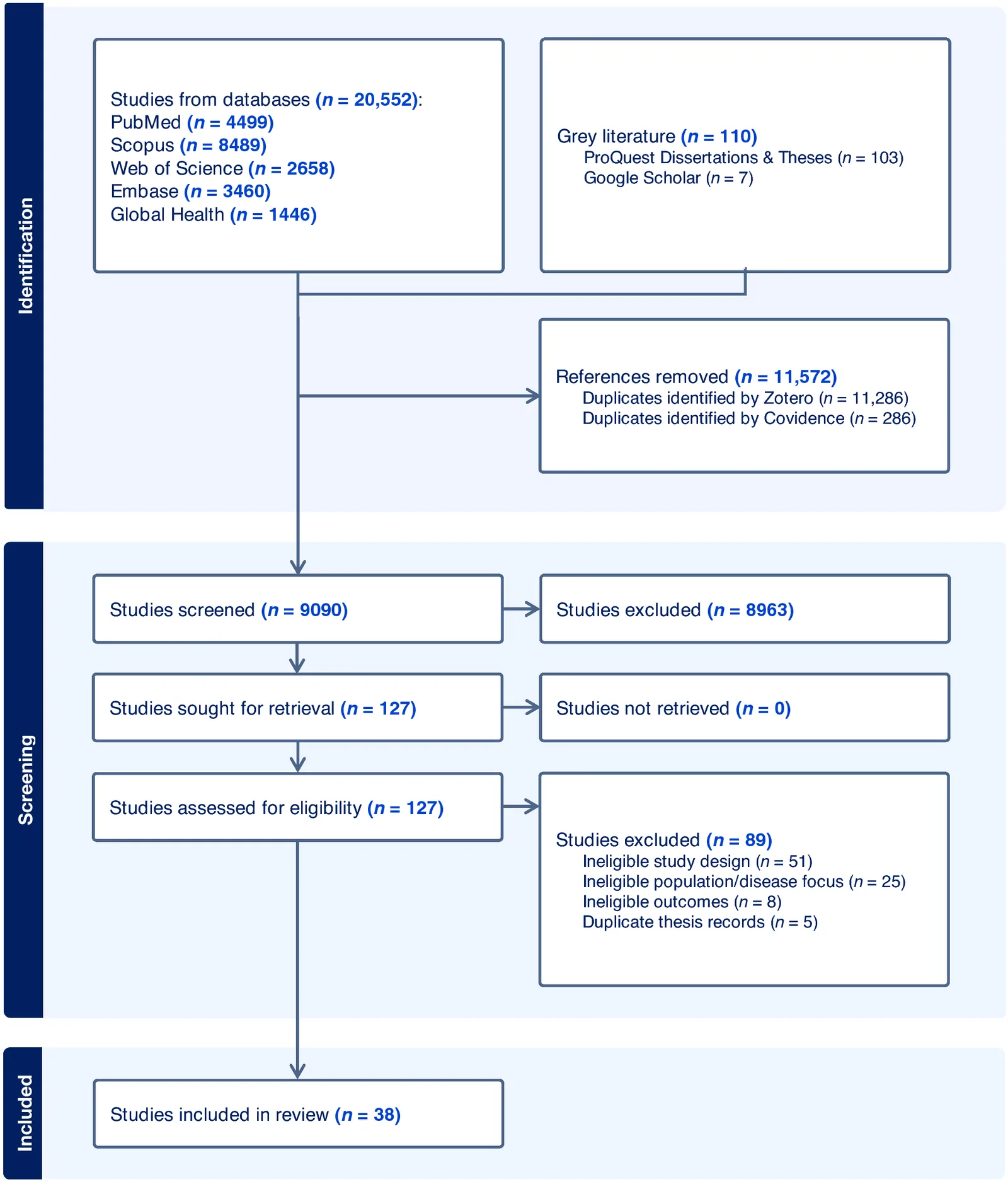
PRISMA (Preferred Reporting Items for Systematic Reviews and Meta-Analyses) 2020 flow diagram illustrating the identification, deduplication, screening, eligibility assessment, and inclusion of studies developing or validating prediction and forecasting models for childhood diarrhoeal disease

We identified 20,662 records, including 20,552 from bibliographic databases (PubMed n=4499; Scopus n=8489; Web of Science n=2658; Embase n=3460; Global Health n=1446) and 110 from grey literature sources (ProQuest Dissertations & Theses n=103; Google Scholar n=7). After removing 11,572 duplicates (Zotero $n=11{,}286$; Covidence n=286), 9,090 unique records were screened by title and abstract, and 8,963 were excluded. We assessed 127 full-text reports and excluded 89 for the following reasons: no predictive or forecasting modelling (n=51), ineligible population or disease focus (n=25), ineligible outcomes (n=8), and duplicate/overlapping publications such as thesis-related (n=5). A detailed list of excluded studies with reasons for exclusion is provided in Additional file 7. In total, 38 studies were included. The study selection process is shown in Fig. 1.

### Characteristics of included studies

**Fig. 2:**
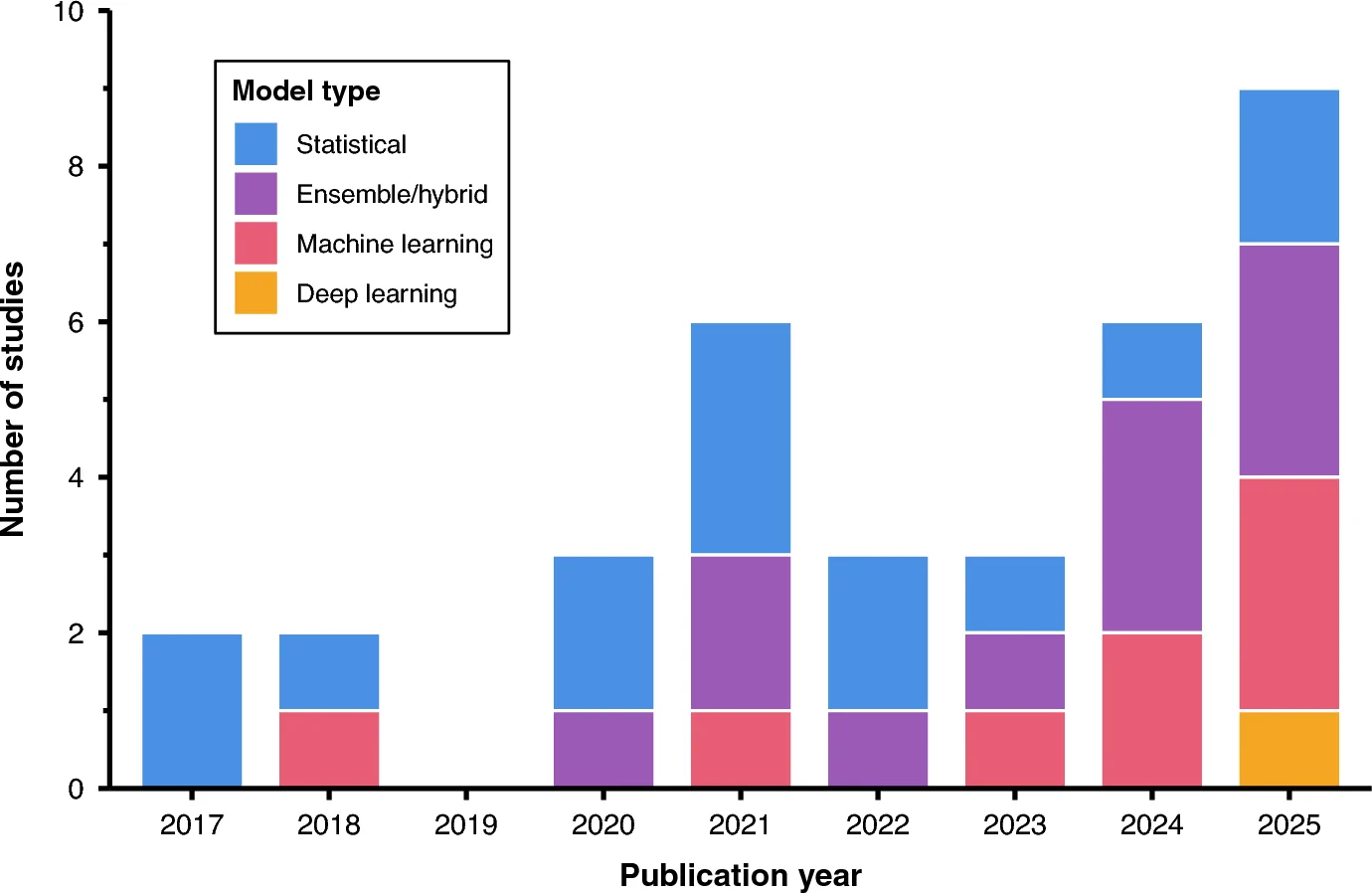
Temporal trends in childhood diarrhoea prediction modelling studies published between 2017 and 2025. *Note:* Prior to 2017, only four studies were identified

The 38 included studies were published between 2000 and 2025, with a marked increase in publication frequency after 2020, during which 78.9% of the studies were published. As shown in Fig. 2, this surge was accompanied by a distinct methodological shift from traditional statistical frameworks towards machine learning and ensemble approaches. An overview of key study characteristics is presented in Table 2, with detailed information and references provided in the Additional file 4.

**Table 2:** Summary of included studies by modelling approach (*n* = 38)

Modelling approach	No. of studies, *n* (%)	Typical predicted outcomes	Common predictor domains/data sources	Validation and implementation-relevant reporting
Statistical models	18 (47.4)	Mostly population-level incidence or case counts, trends, and projections	Frequently environmental or climatic and seasonality or time terms; surveillance, meteorology or remote sensing, and routine health information systems	Validation often emphasised in-sample fit and diagnostics; out-of-sample forecasting was uncommon
Ensemble and hybrid models	11 (28.9)	Individual-level etiology and risk prediction; also early-warning or forecasting in some studies	Often multi-domain predictors combining clinical or demographic and environmental information; multi-site platforms common	Internal resampling was common; a subset used cross-site or independent-cohort validation
Machine learning models	8 (21.1)	Individual-level diarrhoea status or risk stratification and pathogen-related classification; some incidence estimation	Commonly socio-demographic or household and clinical or anthropometric predictors; occasional environmental and genomic features	Train/test splits and cross-validation were common; external validation was uncommon; calibration was infrequently reported
Deep learning models	1 (2.6)	Individual-level clinical prediction or classification	Clinical and demographic variables	Validation was reported, but concerns about overfitting and limited reproducibility were substantial due to very small sample size

#### Geographic distribution and data sources

Studies spanned Asia, Africa, Latin America and the Middle East. Among single-country studies, China contributed the largest number with five studies, representing 20.0% of single-country studies and 13.2% of all included studies. Ethiopia followed with three studies, accounting for 12.0% of single-country studies and 7.9% of all included studies. Bangladesh, Brazil, Botswana, and Indonesia each contributed two studies, corresponding to 8.0% of single-country studies and 5.3% of all included studies. Additional single-country studies were conducted in Thailand, Israel, Mexico, Nepal, Uganda, Nigeria, Kenya, Mozambique, and India, with each country contributing one study, representing 4.0% of single-country studies and 2.6% of all included studies. Thirteen studies, representing 34.2% of the 38 included studies, were multi-country or global. The distribution of studies by country and region is summarized in Table 3. These studies commonly relied on harmonised or multi-site datasets, including five studies from the Global Enteric Multicenter Study (GEMS) and five from the Demographic and Health Surveys (DHS). Two studies used data from the Global Burden of Disease (GBD), one study used MAL-ED, while others utilised pooled multi-country individual participant datasets or global pathogen sequence repositories.

**Table 3:** Distribution of included studies by country or region

Country / region	Number of studies	Percentage (%)
Multi-country / global studies		
Multi-country or global	13	34.2
Single-country studies		
China	5	13.2
Ethiopia	3	7.9
Bangladesh	2	5.3
Brazil	2	5.3
Botswana	2	5.3
Indonesia	2	5.3
Thailand	1	2.6
Israel	1	2.6
Mexico	1	2.6
Nepal	1	2.6
Uganda	1	2.6
Nigeria	1	2.6
Kenya	1	2.6
Mozambique	1	2.6
India	1	2.6
Total	**38**	**100**.**0**

#### Populations and predictor characteristics

Most studies focused on children under five years of age, accounting for 73.7% of the 38 included studies. Ten studies included broader age ranges but reported outcomes relevant to childhood diarrhoea or provided extractable evidence applicable to children. Predictor selection varied widely across studies, covering domains such as socio-demographic and household characteristics, environmental and climatic factors, child-specific clinical factors, and genetic or genomic features. A total of eighty-two unique predictor variables were identified, with full details provided in Additional file 5.

#### Modelling approaches and predictor utilisation

Based on the primary modelling framework used for final prediction or inference, 18 studies used statistical models [52–69], 11 applied ensemble or hybrid approaches [8, 70–79], 8 used machine-learning models [80–87], and 1 used deep learning [88], as summarised in Additional file 6. Predictors from multiple domains were frequently incorporated: environmental and climatic factors in 18 studies, socio-demographic and household characteristics in 23 studies, child-specific biological or clinical factors in 16 studies, and genetic or genomic features in one study. Many predictors used in high-performing models, such as detailed clinical features or high-resolution environmental data, may not be routinely available in surveillance systems, raising concerns about real-world applicability.

### Results of individual studies

#### Individual-level prediction and classification

Individual-level studies examined a range of decision-relevant outcomes, including etiological classification, pathogen-specific infection, mortality risk, persistent diarrhoea, and clinical classification. Performance reporting most commonly focused on discrimination and classification measures, including the area under the receiver operating characteristic curve, sensitivity, specificity, accuracy, and F-scores, while calibration was reported inconsistently. Validation strategies varied, with many studies relying on internal resampling and a smaller subset reporting geographic, temporal, or independent-cohort validation.

These observations align with the conceptual framework (Table 1), where individual-level models are expected to demonstrate external validation and calibration to support reliable clinical or decision-focused applications.

#### Population-level forecasting and early warning

Population-level studies modelled aggregated surveillance outcomes using time-series, spatiotemporal, mechanistic-statistical hybrid, and other forecasting frameworks. Evaluation approaches included residual diagnostics and information criteria within statistical models, as well as error-based measures and retrospective comparisons of forecasts against observed data. However, evaluation designs were often not truly out-of-sample, and forecasting horizons and reporting practices varied substantially.

The remaining four studies comprised pathogen-oriented or other non-standard modelling categories and were summarised descriptively within the final appraisal framework. Consistent with Table 1, these models should ideally be assessed using out-of-sample testing to support operational applications such as outbreak detection or short-term resource planning.

### Risk of bias and methodological quality

To align quality appraisal with modelling objectives, we stratified the 38 included studies into three categories. Fifteen individual-level clinical prediction models were assessed using PROBAST. Nineteen population-level forecasting studies were appraised using a tailored qualitative framework, and the remaining 4 studies were classified as other modelling categories and summarised descriptively.

#### Risk of bias in individual-level models (PROBAST)

**Fig. 3:**
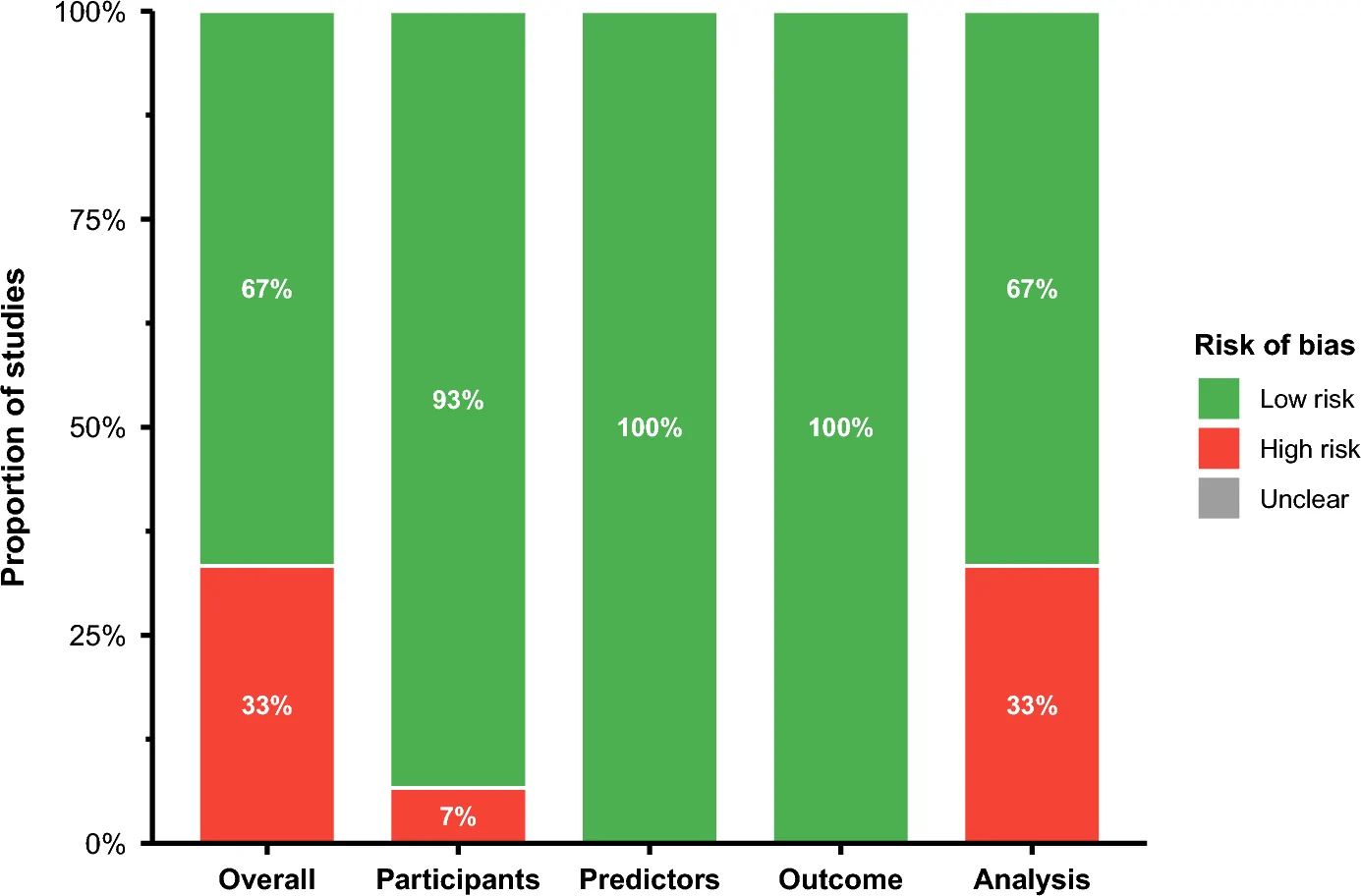
Overall risk of bias assessment of PROBAST-eligible individual-level prediction models (n = 15)

**Fig. 4:**
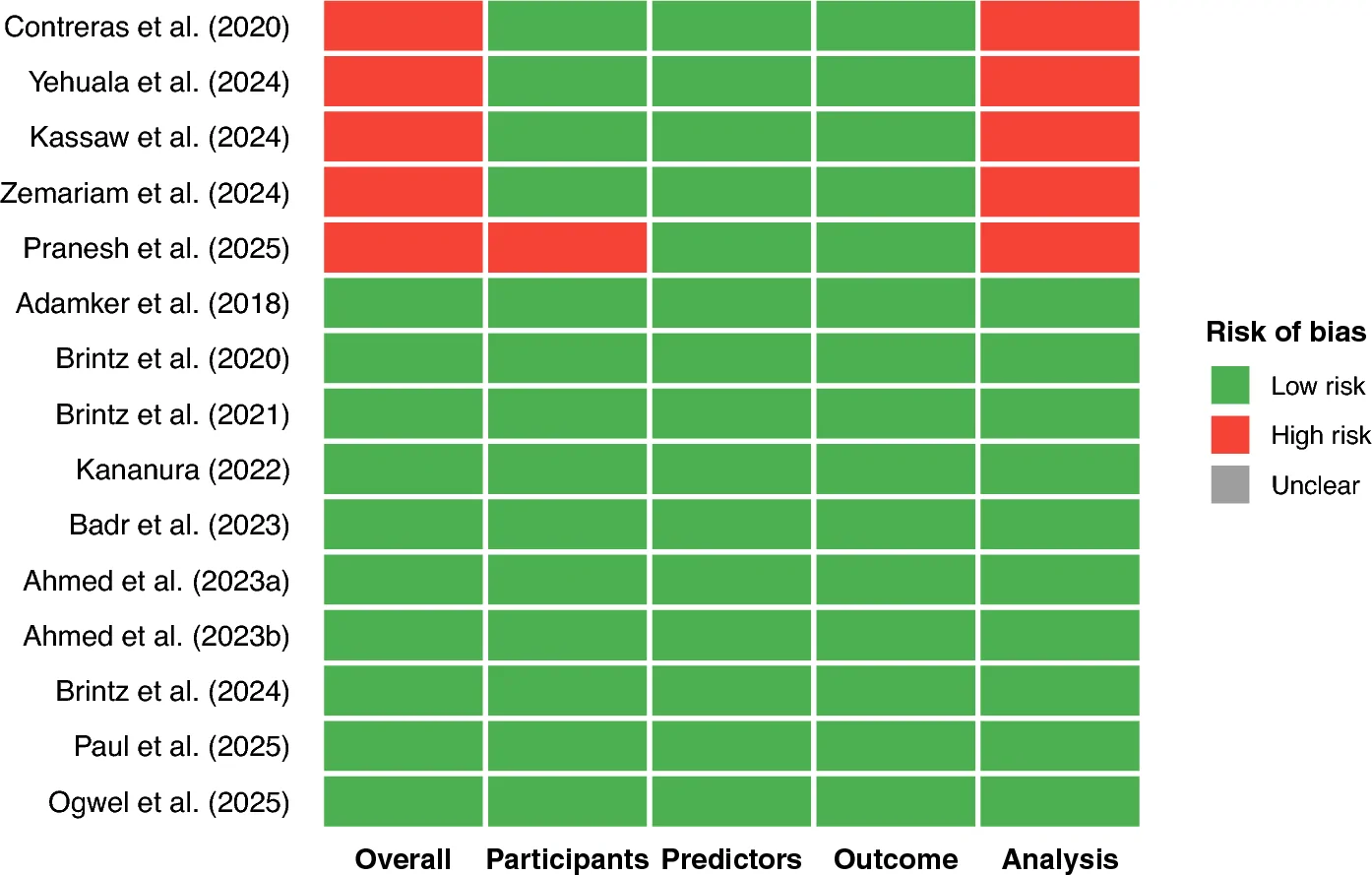
PROBAST traffic-light plot showing domain-level risk of bias judgements for PROBAST-eligible individual-level prediction models (n = 15)

PROBAST was applied to the 15 individual-level multivariable prediction models, representing 39.5% of all included studies. Of the 15 studies assessed, 10 were judged to have a low overall risk of bias and 5 were judged to have a high overall risk of bias. No study was classified as having unclear overall risk. Overall and domain-level judgements are shown in Fig. 3 and Fig. 4. Full details of the risk of bias assessment, including responses to signalling questions for each domain, are available in Additional file 8.

At the domain level, 14 of 15 studies were rated as low risk in the Participants domain, all 15 studies were rated as low risk in the Predictors and Outcome domains, and 5 studies were rated as high risk in the Analysis domain. High-risk judgements in the Analysis domain were driven primarily by lack of formal internal validation[58], potential overoptimism related to class-balancing procedures applied before evaluation[82, 84, 85], and severe overfitting risk in an extremely small deep-learning dataset[88]. In addition, five studies reported external or quasi-external validation using geographic, temporal, or independent-cohort approaches[70, 72, 75, 76, 86], and six studies reported calibration-related metrics[70, 72, 74–77].

#### Qualitative appraisal of population-level forecasting studies

Nineteen population-level time-series and spatiotemporal forecasting studies were appraised qualitatively using the predefined criteria described in the Methods section. These criteria comprised data completeness and temporal resolution, handling of seasonality and non-stationarity, use of true out-of-sample temporal validation, and appropriateness of forecasting performance metrics. The study-level basis for this appraisal is presented in Additional file 10. Among the 19 studies, three reported explicit out-of-sample temporal forecasting evaluations [68, 71, 78]. Forecasting performance was most commonly assessed using in-sample diagnostics, residual analyses, sensitivity analyses, or model-fit measures. A smaller subset of studies additionally reported forecasting-oriented performance metrics, including MAE, RMSE, forecast accuracy measures, MAPE, or related error-based statistics [8, 66, 68, 71, 78, 83].

### Synthesis of findings

Across the 38 included studies, publication frequency increased after 2020, with 30 studies (78.9%) published during this period. Eighteen studies used statistical models, 11 used ensemble or hybrid approaches, 8 used machine-learning models, and 1 used deep learning. Individual-level studies examined outcomes such as diarrhoeal aetiology, pathogen-specific infection, mortality, diarrhoeal status, recovery, and longer-duration or persistent diarrhoea, whereas population-level studies focused on incidence, hospitalisation, outbreak occurrence, temporal trends, and burden projections.

Among the 15 PROBAST-assessed studies, 10 were rated as low overall risk of bias and 5 as high overall risk of bias. Among the 19 population-level forecasting studies, 3 reported explicit out-of-sample temporal validation, while 4 reported clearer predictive validation strategies. Study-level appraisal details for forecasting studies are provided in Additional file 10.

Validation and reporting practices varied across the included studies. Five of the 15 individual-level studies reported external or quasi-external validation[70, 72, 75, 76, 86], and six reported calibration-related metrics[70, 72, 74–77].

## Discussion

This systematic review synthesised the evidence on predictive modelling approaches for childhood diarrhoeal disease and examined their methodological quality, validation practices, and appraisal needs in relation to modelling objective. We identified 38 eligible studies, with a marked increase since 2020, reflecting growing interest in data-driven approaches for clinical prediction, early warning, and public health planning. Despite this growth, substantial heterogeneity remained across the literature, particularly in relation to model classification, validation standards, and the appraisal frameworks used to evaluate model quality and implementation potential.

### Principal findings

Several principal findings emerged from this review. First, the literature expanded rapidly after 2020 and became increasingly methodologically diverse. Although statistical models remain widely used, more recent studies have adopted machine learning, ensemble, and hybrid approaches, often using richer and more heterogeneous datasets. The increasing use of harmonised multi-country data platforms is also notable and reflects growing attention to capturing variation in disease drivers across diverse low- and middle-income settings.

Second, this expansion in modelling diversity was not accompanied by equally consistent validation and reporting practices. Across the included studies, models addressing very different questions were often presented under the broad umbrella of “prediction”, despite differences in unit of analysis, intended use, and evidentiary requirements. Individual-level models, population-level forecasting models, and related pathogen- or classification-oriented studies therefore contributed to a common evidence base, but not to a single methodological category.

Third, the review suggests that the central challenge in this field is no longer the absence of modelling activity, but the need to align methodological expectations with modelling purpose. In this respect, the main contribution of the present review is not only to catalogue predictive approaches, but also to clarify how modelling objective shapes the interpretation of performance, credibility, and practical usefulness.

### Model classification and modelling objectives

A central finding of this review is that childhood diarrhoeal modelling studies should not be interpreted as a single methodological class. Individual-level prediction models, population-level forecasting models, and a smaller subset of pathogen- or classification-oriented studies differ in their intended use, the scale at which predictions are generated, and the decisions they are designed to inform [22, 23]. Individual-level models in this review mainly targeted child-level classification or prognosis, including diarrhoeal aetiology, pathogen-specific infection, mortality, diarrhoeal status, and longer-duration diarrhoea [70, 72, 74–76, 86]. By contrast, population-level studies focused on aggregated outcomes such as incidence, hospitalisation, temporal trends, outbreak occurrence, and burden projection [52, 53, 56, 65, 66, 68, 69, 78].

A smaller subset of studies occupied a related but distinct position within the evidence base, including pathogen/genomic classification and other non-standard modelling categories [60, 67, 81, 87]. These studies were relevant to the broader landscape of childhood diarrhoeal modelling, but their outputs differed from both child-level risk prediction and population-level forecasting. For this reason, they are better interpreted as adjacent forms of predictive evidence than as directly comparable alternatives within the same appraisal stratum. Making these distinctions explicit helps maintain the methodological focus of the review while avoiding the assumption that all model-based studies address the same prediction task.

### Validation practices across modelling objectives

A second major finding is that validation remained a consistent weakness across modelling objectives, although the nature of this weakness differed between individual-level and population-level studies. In individual-level models, the main limitations concerned external validation, calibration reporting, and evidence of transportability beyond the original development setting. In population-level forecasting studies, the more relevant issue was whether models were evaluated on genuinely held-out future periods using forecasting-appropriate strategies and metrics, rather than simply demonstrating good fit to historical data.

Among the individual-level studies, only a minority reported external or quasi-external validation across independent sites, regions, or time periods[70, 72, 75, 76, 86]. Calibration was also reported inconsistently, despite its importance for determining whether predicted probabilities correspond to observed risks[25]. Most studies instead emphasised discrimination metrics, such as the area under the curve (AUC), while giving less attention to calibration, threshold performance, or model updating in new settings. These patterns are not unique to childhood diarrhoeal modelling and are consistent with broader concerns in prediction research regarding over-optimism and limited transportability when models are evaluated primarily within development datasets [23, 89]. Reporting standards such as TRIPOD and TRIPOD-AI remain highly relevant in this context, because they make it easier to judge whether a model can be interpreted, reproduced, and applied beyond its original analytical setting [22, 90].

The population-level forecasting literature showed a different but equally important validation gap. Only a small number of studies reported explicit out-of-sample temporal forecasting evaluations[68, 71, 78], whereas many others relied on in-sample diagnostics, residual analyses, sensitivity analyses, or internal fit measures. This distinction is especially important for forecasting models, because accurate reconstruction of historical patterns does not necessarily indicate that a model performs well when applied to future periods. The same principle applies to the choice of performance measures. A smaller subset of studies reported forecasting-oriented metrics such as MAE, RMSE, MAPE, forecast accuracy measures, or related error-based statistics[8, 66, 68, 71, 78, 83], whereas others relied more heavily on inferential statistics, goodness-of-fit measures, or descriptive diagnostics. Taken together, these findings suggest that validation should be interpreted relative to modelling objective rather than treated as a single generic requirement across all predictive studies.

A small number of studies nevertheless showed that more rigorous validation and reporting are feasible. These studies typically used larger, harmonised, or multi-site datasets; evaluated performance across independent settings or future periods; and provided more complete information on discrimination, calibration, or forecasting performance[68, 70–72, 75, 76, 78, 86, 91, 92]. Their presence in the literature is important because it shows that stronger methodological standards are achievable even in settings where data and implementation constraints are substantial.

### Appraisal framework and methodological quality

The findings of this review support the need for appraisal frameworks that are aligned with modelling objective rather than applied uniformly across all model types. One of the central methodological challenges in this literature is that studies using different prediction targets and decision contexts are often judged using inconsistent or implicitly mixed standards. By separating appraisal strata according to modelling objective, the present review aimed to make these methodological distinctions more explicit.

For individual-level models, PROBAST provided a structured approach to evaluating methodological quality across participants, predictors, outcomes, and analysis. This was particularly useful for distinguishing studies with more complete reporting and stronger validation practices from those with more limited analytical robustness. Importantly, the use of official PROBAST categories helped preserve interpretive clarity while avoiding additional grading layers that are not part of the original framework. At the same time, applying PROBAST to more complex machine learning models was not always straightforward and required careful judgement, especially when reporting of feature selection, hyperparameter tuning, and model optimisation procedures was incomplete [39, 89, 93].

For population-level forecasting studies, however, a different appraisal lens was required. These studies are not primarily evaluated by whether they estimate risk for an individual child, but by how they handle temporality, seasonality, non-stationarity, data structure, and future-period prediction. The qualitative appraisal used in this review was therefore intended to improve transparency regarding the methodological basis of reported forecasting performance without imposing an individual-level clinical prediction framework on studies for which it was not designed. In this respect, the review reinforces that methodological quality must be interpreted in relation to intended model use.

This issue is closely related to implementation readiness. Across both appraisal strata, the practical usefulness of a model depended not only on its apparent performance, but also on whether the study reported sufficient information to support reproducibility, contextual interpretation, and informed adaptation. From this perspective, implementation readiness is best understood as the intersection of methodological quality, validation practice, reporting transparency, and compatibility with the intended decision context. Models that appear technically sophisticated but are insufficiently validated or poorly reported may therefore contribute less to practice than simpler models with clearer evaluation and greater transparency [94].

### Geographic distribution and predictor patterns

Although the central theme of this review is methodological, the geographic distribution and predictor composition of the evidence base also help explain how these models have been developed and where their likely value lies. The included studies were concentrated largely in low- and middle-income settings, where the burden of childhood diarrhoeal disease is disproportionately high; however, representation remained uneven across regions and surveillance environments. A relatively small number of countries and data platforms contributed a substantial proportion of the available evidence, while some high-burden settings remained under-represented.

This pattern has methodological as well as epidemiological implications. Studies based on harmonised multi-country datasets or large surveillance platforms may offer broader comparative value, whereas models developed in highly specific local data environments may be more difficult to transport across settings with different health systems, climatic exposures, sanitation conditions, or pathogen ecologies. In this sense, geographic concentration is relevant not only as a descriptive feature of the literature, but also as a factor shaping model generalisability and implementation potential [95].

Predictor selection also differed systematically across modelling objectives. Population-level forecasting studies relied more heavily on climatic, environmental, and surveillance-derived temporal indicators, reflecting their use in early warning and incidence prediction. Individual-level models more often used clinical, demographic, and household-level predictors, consistent with case classification and prognosis. Socioeconomic and WASH-related variables appeared across both traditions, but their role varied according to the scale and purpose of the modelling task. These differences reinforce that predictor choice is not merely a technical detail; it reflects the operational question the model is intended to answer.

For early-warning and forecasting applications in particular, the practical value of predictors depends not only on statistical association, but also on routine availability, timeliness, and compatibility with existing surveillance workflows. This is especially important in resource-constrained settings, where models intended for operational use must rely on variables that can be collected, updated, and interpreted within real-world public health systems [96]. Closer alignment between predictor design and implementation context may therefore be as important as improvements in algorithmic complexity.

### Strengths and limitations of this review

This review has several strengths. It was based on a comprehensive search strategy that included multiple bibliographic databases and grey literature sources, thereby reducing the likelihood that relevant studies were missed solely because of publication pathway. A further strength is the explicit separation of studies according to modelling objective and appraisal stratum. Applying PROBAST to individual-level prediction models while appraising population-level forecasting studies with a complementary qualitative framework allowed us to evaluate methodological quality across a heterogeneous evidence base without imposing a single inappropriate standard across all studies. To our knowledge, relatively few previous reviews in childhood diarrhoeal modelling have taken this dual approach.

Several limitations should be considered. First, this review relied on published reports and supplementary materials rather than primary datasets, limiting our ability to verify unreported analytical details or recalculate model performance. Second, marked heterogeneity in populations, outcomes, predictors, modelling approaches, validation procedures, and reported performance measures precluded quantitative synthesis and limited direct comparison across studies. Third, restricting inclusion to English-language publications may have introduced language bias and excluded relevant evidence from some high-burden settings. In addition, our Google Scholar screening was limited to the first 100 relevance-ranked records, so some relevant grey literature may not have been captured. A further limitation concerns quality appraisal. Although structured appraisal tools improve consistency, their application to complex AI-oriented studies remains constrained by the completeness and transparency of reporting in the original publications; some methodological details were not described in sufficient depth to support fully confident judgements [39]. Finally, although this review retained genomic and pathogen-focused modelling studies within the broader evidence base, these studies were not examined in the same depth as the individual-level prediction and population-level forecasting literature. As this area continues to expand, future reviews could provide a more dedicated and method-specific synthesis of these models.

Overall, this review suggests that the field of childhood diarrhoeal predictive modelling has advanced substantially in technical range, but that its interpretation depends on clearer distinction between modelling objectives, more appropriate validation practices, and appraisal approaches matched to intended use. Making these distinctions more explicit may help the literature move from methodological diversity alone towards more credible, transparent, and decision-relevant evidence.

## Conclusions

Given its high, largely preventable burden and heterogeneous determinants, prediction modelling for childhood diarrhoeal disease has expanded rapidly, spanning individual-level prediction, population-level forecasting, and pathogen- or classification-oriented models. Although some studies reported stronger validation practices, including external validation, calibration assessment, or temporal out-of-sample evaluation, these remained uncommon, limiting confidence in transportability and implementation readiness. Future progress will depend on better alignment between modelling objectives, validation strategy, appraisal framework, and transparent reporting.

## Supplementary Information

Supplementary file 1. PRISMA 2020 checklist. Completed checklist documenting compliance with PRISMA 2020 guidelines.

Supplementary file 2. Conceptual framework table. Detailed conceptual framework linking modelling objectives to validation requirements and public health goals.

Supplementary file 3. Search strategy. Full reproducible search strategies for all searched databases.

Supplementary file 4. Characteristics of included studies. Detailed table of study characteristics, populations, and outcomes for the 38 included studies.

Supplementary file 5. Predictor variables summary. Comprehensive list of the predictor variables identified and their domain classification.

Supplementary file 6. Modelling approaches. Summary of statistical, machine learning, and hybrid modelling approaches used in the included studies.

Supplementary file 7. Excluded full text studies. List of excluded full-text articles with reasons for exclusion.

Supplementary file 8. PROBAST risk of bias assessment. Detailed domain-level risk of bias judgements for individual-level prediction models.

Supplementary file 9. Appraisal crosswalk of included studies. Crosswalk table mapping each included study to its modelling objective, appraisal stratum, and corresponding appraisal approach used in this review.

Supplementary file 10. Qualitative appraisal of population-Level forecasting studies. Study-level qualitative appraisal of population-level forecasting studies based on predefined epidemiological forecasting criteria, including temporal data structure, handling of seasonality and non-stationarity, use of out-of-sample forecasting evaluation, and appropriateness of reported forecasting performance measures.

## Data Availability

No datasets were generated or analysed during the current study.

## Code availability

The analytical code used in this study is available from the corresponding author upon reasonable request.

## References

[1] GBD Diarrhoeal Disease and Enteric Infectious Diseases Collaborators. Global burden of enteric infectious diseases, diarrhoeal diseases, and corresponding aetiologies, 1990–2023: a systematic analysis for the Global Burden of Disease Study. Lancet Infect Dis. 2026. 10.1016/S1473-3099(26)00194-5. 42229499

[2] Lanata CF, Fischer-Walker CL, Olascoaga AC, Torres CX, Aryee MJ, Black RE, et al. Global causes of diarrheal disease mortality in children<5 years of age: a systematic review. PLoS ONE. 2013;8(9):e72788. 10.1371/journal.pone.0072788. PMC3762858 24023773

[3] Troeger C, Colombara DV, Rao PC, Khalil IA, Brown A, Brewer TG, et al. Global disability-adjusted life-year estimates of long-term health burden and undernutrition attributable to diarrhoeal diseases in children younger than 5 years. Lancet Glob Health. 2018;6(3):e255–69. 10.1016/S2214-109X(18)30045-7. PMC5861379 29433665

[4] Reiner RC Jr, Graetz N, Casey DC, Troeger C, Garcia GM, Mosser JF, et al. Variation in childhood diarrheal morbidity and mortality in Africa, 2000–2015. N Engl J Med. 2018;379(12):1128–38. 10.1056/NEJMoa1716766 PMC6078160 30231224

[5] Li Z, Meng F, Wu B, Kong D, Geng M, Qiu X, et al. Reviewing the progress of infectious disease early warning systems and planning for the future. BMC Public Health. 2024;24(1):3080. 10.1186/s12889-024-20537-2 PMC11542453 39511577

[6] Ogwel B, Mzazi V, Nyawanda BO, Otieno G, Omore R. Predictive modeling for infectious diarrheal disease in pediatric populations: a systematic review. Learn Health Syst. 2024;8(1):e10382. 10.1002/lrh2.10382 PMC10797570 38249852

[7] Colston JM, Zaitchik B, Kang G, Yori PP, Ahmed T, Lima A, et al. Use of earth observation-derived hydrometeorological variables to model and predict rotavirus infection (MAL-ED): a multisite cohort study. Lancet Planet Health. 2019;3(6):e248–58. 10.1016/S2542-5196(19)30084-1 PMC6650544 31229000

[8] Alexander KA, Heaney AK, Shaman J. Hydrometeorology and flood pulse dynamics drive diarrheal disease outbreaks and increase vulnerability to climate change in surface-water-dependent populations: a retrospective analysis. PLoS Med. 2018;15(11):e1002688. 10.1371/journal.pmed.1002688 PMC6224043 30408029

[9] Delahoy MJ, Cárcamo C, Huerta A, Lavado W, Escajadillo Y, Ordoñez L, et al. Meteorological factors and childhood diarrhea in Peru, 2005–2015: a time series analysis of historic associations, with implications for climate change. Environ Health. 2021;20(1):22. 10.1186/s12940-021-00703-4 PMC7913169 33637108

[10] Dimitrova A, Gershunov A, Levy MC, Benmarhnia T. Uncovering social and environmental factors that increase the burden of climate-sensitive diarrheal infections on children. Proc Natl Acad Sci USA. 2023;120(3):e2119409120. 10.1073/pnas.2119409120 PMC9934300 36623190

[11] Abdullahi T, Nitschke G, Sweijd N. Predicting diarrhoea outbreaks with climate change. PLoS ONE. 2022;17(4):e0262008. 10.1371/journal.pone.0262008 PMC9017952 35439258

[12] Mellor JE, Levy K, Zimmerman J, Elliott M, Bartram J, Carlton E, et al. Planning for climate change: the need for mechanistic systems-based approaches to study climate change impacts on diarrheal diseases. Sci Total Environ. 2016;548:82–90. 10.1016/j.scitotenv.2015.12.087 PMC4818006 26799810

[13] Palagyi A, Marais BJ, Abimbola S, Topp SM, McBryde ES, Negin J. Health system preparedness for emerging infectious diseases: a synthesis of the literature. Glob Public Health. 2019;14(12):1847–68. 10.1080/17441692.2019.1614645 31084412

[14] Meki CD, Ncube EJ, Voyi K. Frameworks for mitigating the risk of waterborne diarrheal diseases: a scoping review. PLoS ONE. 2022;17(12):e0278184. 10.1371/journal.pone.0278184 PMC9733858 36490262

[15] Carlton EJ, Woster AP, DeWitt P, Goldstein RS, Levy K. A systematic review and meta-analysis of ambient temperature and diarrhoeal diseases. Int J Epidemiol. 2016;45(1):117–30. 10.1093/ije/dyv296 PMC4881833 26567313

[16] Watts N, Amann M, Arnell N, Ayeb-Karlsson S, Beagley J, Belesova K, et al. The 2020 report of the lancet countdown on health and climate change: responding to converging crises. Lancet. 2021;397(10269):129–70. 10.1016/S0140-6736(20)32290-X PMC7616803 33278353

[17] Wang P, Zhang W, Wang H, Shi C, Li Z, Wang D, et al. Predicting the incidence of infectious diarrhea with symptom surveillance data using a stacking-based ensembled model. BMC Infect Dis. 2024;24(1):265. 10.1186/s12879-024-09138-x PMC10898154 38408967

[18] Tosepu R, Ningsi NY. Forecasting of diarrhea disease using ARIMA model in Kendari City, Southeast Sulawesi Province, Indonesia. Heliyon. 2024;10(22):e40247. 10.1016/j.heliyon.2024.e40247. PMC11600073 39605824

[19] Buczak AL, Koshute PT, Babin SM, Feighner BH, Lewis SH. A data-driven epidemiological prediction method for dengue outbreaks using local and remote sensing data. BMC Med Inform Decis Mak. 2012;12(1):124. 10.1186/1472-6947-12-124 PMC3534444 23126401

[20] Pezanowski S, Koua EL, Okeibunor JC, Gueye AS. Predictors of disease outbreaks at continental-scale in the African region: insights and predictions with geospatial artificial intelligence using earth observations and routine disease surveillance data. Digit Health. 2024;10:20552076241278940. 10.1177/20552076241278939 PMC11539184 39507013

[21] Sutton RT, Pincock D, Baumgart DC, Sadowski DC, Fedorak RN, Kroeker KI. An overview of clinical decision support systems: benefits, risks, and strategies for success. NPJ Digit Med. 2020;3(1):17. 10.1038/s41746-020-0221-y PMC7005290 32047862

[22] Collins GS, Reitsma JB, Altman DG, Moons KG. Transparent reporting of a multivariable prediction model for individual prognosis or diagnosis (TRIPOD): the TRIPOD statement. Br J Surg. 2015;102(3):148–58. 10.1002/bjs.9736 25627261

[23] Moons KG, Kengne AP, Woodward M, Royston P, Vergouwe Y, Altman DG. Risk prediction models: I. development, internal validation, and assessing the incremental value of a new (bio) marker. Heart. 2012;98(9):683–90. 10.1136/heartjnl-2011-301246 22397945

[24] Riley RD, Ensor J, Snell KIE, Debray TPA, Altman DG, Moons KGM, et al. External validation of clinical prediction models using big datasets from e-health records or IPD meta-analysis: opportunities and challenges. BMJ. 2019;365:l4379. 10.1136/bmj.l4379. PMC4916924 27334381

[25] Van Calster B, McLernon DJ, Van Smeden M, Wynants L, Steyerberg EW. Calibration: the achilles heel of predictive analytics. BMC Med. 2019;17(1):230. 10.1186/s12916-019-1466-7 PMC6912996 31842878

[26] Moons KG, Wolff RF, Riley RD, Whiting PF, Westwood M, Collins GS, et al. PROBAST: a tool to assess risk of bias and applicability of prediction model studies: explanation and elaboration. Ann Intern Med. 2019;170(1):W1–33. 10.7326/M18-1377 30596876

[27] Moons KG, Damen JA, Kaul T, Hooft L, Andaur Navarro C, Dhiman P, et al. PROBAST+AI: an updated quality, risk of bias, and applicability assessment tool for prediction models using regression or artificial intelligence methods. BMJ. 2025;388:e082505. 10.1136/bmj-2024-082505. PMC11931409 40127903

[28] Bracher J, Ray EL, Gneiting T, Reich NG. Evaluating epidemic forecasts in an interval format. PLoS Comput Biol. 2021;17(2):e1008618. 10.1371/journal.pcbi.1008618 PMC7880475 33577550

[29] Topol EJ. High-performance medicine: the convergence of human and artificial intelligence. Nat Med. 2019;25(1):44–56. 10.1038/s41591-018-0300-7 30617339

[30] United Nations. Transforming our world: the 2030 Agenda for Sustainable Development. New York: United Nations; 2015. Accessed 17 July 2026. Available from: https://sdgs.un.org/2030agenda.

[31] National Institute for Health and Care Research.: PROSPERO: international prospective register of systematic reviews. Accessed 17 July 2026. https://www.crd.york.ac.uk/prospero/.

[32] Page MJ, McKenzie JE, Bossuyt PM, Boutron I, Hoffmann TC, Mulrow CD, et al. The PRISMA 2020 statement: an updated guideline for reporting systematic reviews. BMJ. 2021;372:n71. 10.1136/bmj.n71. PMC8005924 33782057

[33] Rethlefsen ML, Kirtley S, Waffenschmidt S, Ayala AP, Moher D, Page MJ, et al. PRISMA-S: an extension to the PRISMA statement for reporting literature searches in systematic reviews. Syst Rev. 2021;10(1):39. 10.1186/s13643-020-01542-z PMC7839230 33499930

[34] Corporation for Digital Scholarship.: Zotero. Vienna, VA, USA. Version 7.0.32. Accessed 17 July 2026. https://www.zotero.org/.

[35] Veritas Health Innovation.: Covidence systematic review software. Melbourne, VIC, Australia. Accessed 17 July 2026. https://www.covidence.org/.

[36] Debray TP, Damen JA, Snell KI, Ensor J, Hooft L, Reitsma JB, et al. A guide to systematic review and meta-analysis of prediction model performance. BMJ. 2017;356:i6460. 10.1136/bmj.i6460. 28057641

[37] Bramer WM, Giustini D, De Jonge GB, Holland L, Bekhuis T. De-duplication of database search results for systematic reviews in EndNote. J Med Libr Assoc. 2016;104(3):240–3. 10.3163/1536-5050.104.3.014. PMC4915647 27366130

[38] Moons KG, de Groot JA, Bouwmeester W, Vergouwe Y, Mallett S, Altman DG, et al. Critical appraisal and data extraction for systematic reviews of prediction modelling studies: the CHARMS checklist. PLoS Med. 2014;11(10):e1001744. 10.1371/journal.pmed.1001744 PMC4196729 25314315

[39] Wolff RF, Moons KG, Riley RD, Whiting PF, Westwood M, Collins GS, et al. PROBAST: a tool to assess the risk of bias and applicability of prediction model studies. Ann Intern Med. 2019;170(1):51–8. 10.7326/M18-1376 30596875

[40] R Core Team.: R: a language and environment for statistical computing. Vienna, Austria. Version 4.5.2. Accessed 17 July 2026. https://www.R-project.org/.

[41] Posit Team.: RStudio: integrated development environment for R. Boston, MA, USA. Version 2026.01. Accessed 17 July 2026. https://posit.co/.

[42] Wickham H, Averick M, Bryan J, Chang W, McGowan LD, François R, et al. Welcome to the tidyverse. J Open Source Softw. 2019;4(43):1686.

[43] Wickham H, François R, Henry L, Müller K, Vaughan D. dplyr: a grammar of data manipulation. R package version 1.1.4, Accessed 17 July 2026. https://CRAN.R-project.org/package=dplyr.

[44] Wickham H, Vaughan D, Girlich M. tidyr: tidy messy data. R package version 1.3.1, Accessed 17 July 2026. https://CRAN.R-project.org/package=tidyr.

[45] Wickham H, Seidel D. scales: scale functions for visualization. R package version 1.3.0, Accessed 17 July 2026. https://CRAN.R-project.org/package=scales.

[46] Wickham H, Chang W, Henry L, Pedersen TL, Takahashi K, Wilke C, et al. ggplot2: create elegant data visualisations using the grammar of graphics. R package version 4.0.1. Accessed 17 July 2026. https://CRAN.R-project.org/package=ggplot2.

[47] Pollett S, Johansson MA, Reich NG, Brett-Major D, Del Valle SY, Venkatramanan S, et al. Recommended reporting items for epidemic forecasting and prediction research: the EPIFORGE 2020 guidelines. PLoS Med. 2021;18(10):e1003793. 10.1371/journal.pmed.1003793. PMC8525759 34665805

[48] Pitzer VE, Viboud C, Lopman BA, Patel MM, Parashar UD, Grenfell BT. Influence of birth rates and transmission rates on the global seasonality of rotavirus incidence. J R Soc Interface. 2011;8(64):1584–93. 10.1098/rsif.2011.0062 PMC3177613 21508015

[49] Hyndman RJ, Athanasopoulos G. Forecasting: principles and practice. 2nd ed. Melbourne, Australia: OTexts; 2018. Accessed 17 July 2026. https://otexts.com/fpp2/.

[50] Campbell M, McKenzie JE, Sowden A, Katikireddi SV, Brennan SE, Ellis S, et al. Synthesis without meta-analysis (SWiM) in systematic reviews: reporting guideline. BMJ. 2020;368:l6890. 10.1136/bmj.l6890. PMC7190266 31948937

[51] Popay J, Roberts H, Sowden A, Petticrew M, Arai L, Rodgers M. Guidance on the conduct of narrative synthesis in systematic reviews. ESRC Methods Programme. 2006.

[52] Kale PL, Hinde JP, Nobre FF. Modeling diarrhea disease in children less than 5 years old. Ann Epidemiol. 2004;14(6):371–7. 10.1016/j.annepidem.2003.08.001 15246324

[53] Kale PL, Andreozzi VL, Nobre FF. Time series analysis of deaths due to diarrhoea in children in Rio de Janeiro, Brazil, 1980–1998. J Health Popul Nutr. 2004;22(1):27–33. 15190809

[54] Hashizume M, Armstrong B, Wagatsuma Y, Faruque A, Hayashi T, Sack DA. Rotavirus infections and climate variability in Dhaka, Bangladesh: a time-series analysis. Epidemiol Infect. 2008;136(9):1281–9. 10.1017/S0950268807009776 PMC2870922 17988426

[55] Ardkaew J, Tongkumchum P. Statistical modelling of childhood diarrhea in northeastern Thailand. Southeast Asian J Trop Med Public Health. 2009;40(4):807–15. 19842419

[56] Azage M, Kumie A, Worku A, Bagtzoglou AC, Anagnostou E. Effect of climatic variability on childhood diarrhea and its high risk periods in northwestern parts of Ethiopia. PLoS ONE. 2017;12(10):e0186933. 10.1371/journal.pone.0186933 PMC5658103 29073259

[57] Hasan MA, Mouw C, Jutla A, Akanda AS. Quantification of rotavirus diarrheal risk due to hydroclimatic extremes over South Asia: prospects of satellite-based observations in detecting outbreaks. Geohealth. 2018;2(2):70–86. 10.1002/2017GH000101 PMC7007079 32159010

[58] Contreras JD, Trangucci R, Felix-Arellano EE, Rodríguez-Dozal S, Siebe C, Riojas-Rodríguez H, et al. Modeling spatial risk of diarrheal disease associated with household proximity to untreated wastewater used for irrigation in the Mezquital Valley, Mexico. Environ Health Perspect. 2020;128(7):077002. 10.1289/EHP6443 PMC7340213 32634028

[59] Lu W, Li J, Li J, Ai D, Song H, Duan Z, et al. Short-term impacts of meteorology, air pollution, and internet search data on viral diarrhea infection among children in Jilin Province, China. Int J Environ Res Public Health. 2021;18(21):11615. 10.3390/ijerph182111615 PMC8582928 34770125

[60] Fan X, Xue N, Han Z, Wang C, Ma H, Lu Y. Wavelet transform artificial intelligence algorithm-based data mining technology for norovirus monitoring and early warning. J Healthc Eng. 2021;2021(1):6128260. 10.1155/2021/6128260 PMC8463185 34567483

[61] Huang NN, Zheng H, Li B, Fei GQ, Ding Z, Wang JJ, et al. The short-term effects of temperature on infectious diarrhea among children under 5 years old in Jiangsu, China: a time-series study (2015–2019). Curr Med Sci. 2021;41(2):211–8. 10.1007/s11596-021-2338-x PMC8056199 33877537

[62] Jiwok JC, Adebowale AS, Wilson I, Kancherla V, Umeokonkwo CD. Patterns of diarrhoeal disease among under-five children in Plateau State, Nigeria, 2013–2017. BMC Public Health. 2021;21(1):2086. 10.1186/s12889-021-12110-y PMC8590335 34774002

[63] Kananura RM. Machine learning predictive modelling for identification of predictors of acute respiratory infection and diarrhoea in Uganda’s rural and urban settings. PLOS Glob Public Health. 2022;2(5):e0000430. 10.1371/journal.pgph.0000430 PMC10021828 36962243

[64] Dhimal M, Bhandari D, Karki KB, Shrestha SL, Khanal M, Shrestha RRP, et al. Effects of climatic factors on diarrheal diseases among children below 5 years of age at national and subnational levels in Nepal: an ecological study. Int J Environ Res Public Health. 2022;19(10):6138. 10.3390/ijerph19106138 PMC9140521 35627674

[65] Haque F. Impacts of temperature, diurnal temperature range, heat index and heat wave on diarrhoeal diseases in Dhaka, Bangladesh in the context of climate change: a time-series analysis [PhD thesis]. University College London. London, UK; 2022. Accessed 17 July 2026. https://discovery.ucl.ac.uk/id/eprint/10155876.

[66] Jaya I, Chadidjah A, Andriyana Y, Kristiani F, Fauziah AN. Bayesian hierarchical spatiotemporal modeling for forecasting diarrhea risk among children under 5 in Bandung city. Indonesia Int J Data Netw Sci. 2023;7(4):1551–62.

[67] Faidah DY, Hudzaifa AM, Pontoh RS. Clustering of childhood diarrhea diseases using Gaussian mixture model. Commun Math Biol Neurosci. 2024;2024:10.

[68] Zhou J, Yao Y, Li L, Wang X, Dai T, Cai X, et al. Climatic drivers of infectious diarrheal disease epidemics in China: an empirical dynamic modeling analysis of 21 million cases. J Infect. 2025;91(1):106518. 10.1016/j.jinf.2025.106518 40414566

[69] Liu S, Wu Z, An Q, Wu J, Bai J, Sun W, et al. The burden and etiologies of diarrhea in Asia and its countries from 1990 to 2021 and the forecast to 2040: analyses informed by the global burden of disease study 2021. Front Public Health. 2025;13:1651315. 10.3389/fpubh.2025.1651315 PMC12364947 40843416

[70] Brintz BJ, Howard JI, Haaland B, Platts-Mills JA, Greene T, Levine AC, et al. Clinical predictors for etiology of acute diarrhea in children in resource-limited settings. PLoS Negl Trop Dis. 2020;14(10):e0008677. 10.1371/journal.pntd.0008677 PMC7588112 33035209

[71] Heaney AK, Alexander KA, Shaman J. Ensemble forecast and parameter inference of childhood diarrhea in Chobe District. Botswana Epidemics. 2020;30:100372. 10.1016/j.epidem.2019.100372 PMC7669214 31551173

[72] Brintz BJ, Haaland B, Howard J, Chao DL, Proctor JL, Khan AI, et al. A modular approach to integrating multiple data sources into real-time clinical prediction for pediatric diarrhea. Elife. 2021;10:e63009. 10.7554/eLife.63009 PMC7853717 33527894

[73] Fagbamigbe AF, Uthman AO, Ibisomi L. Hierarchical disentanglement of contextual from compositional risk factors of diarrhoea among under-five children in low- and middle-income countries. Sci Rep. 2021;11(1):8564. 10.1038/s41598-021-87889-2 PMC8058334 33879839

[74] Badr HS, Colston JM, Nguyen NLH, Chen YT, Burnett E, Ali SA, et al. Spatiotemporal variation in risk of Shigella infection in childhood: a global risk mapping and prediction model using individual participant data. Lancet Glob Health. 2023;11(3):e373–84. 10.1016/S2214-109X(22)00549-6 PMC10020138 36796984

[75] Ahmed SM, Brintz BJ, Pavlinac PB, Hossain MI, Khan AI, Platts-Mills JA, et al. Clinical prediction rule to guide diagnostic testing for shigellosis and improve antibiotic stewardship for pediatric diarrhea. Open Forum Infect Dis. 2023;10(3):ofad119. 10.1093/ofid/ofad119 PMC10043128 36998629

[76] Ahmed SM, Brintz BJ, Talbert A, Ngari M, Pavlinac PB, Platts-Mills JA, et al. Derivation and external validation of a clinical prognostic model identifying children at risk of death following presentation for diarrheal care. PLOS Glob Public Health. 2023;3(6):e0001937. 10.1371/journal.pgph.0001937 PMC10298785 37368867

[77] Brintz BJ, Colston JM, Ahmed SM, Chao DL, Zaitchik BF, Leung DT. Assessment and comparison of model estimated and directly observed weather data for prediction of diarrhoea aetiology. Epidemiol Infect. 2024;152:e122. 10.1017/S0950268824001183 PMC11488466 39381928

[78] Yaari R, Galanti M, Zepeda-Tello R, Chicumbe S, Jani I, Cassy A, et al. Infectious disease forecasting to support public health: use of readily available methods to predict malaria and diarrhoeal diseases in Mozambique. J Glob Health. 2025;15:04114. 10.7189/jogh.15.04114 PMC11997905 40214148

[79] Paul S, Rahman M, Dolley A, Saikia K, Singh CS, Mohammed A, et al. A retrospective study using machine learning to develop predictive model to identify rotavirus-associated acute gastroenteritis in children. PeerJ. 2025;13:e19025. 10.7717/peerj.19025 PMC12005185 40247842

[80] Adamker G, Holzer T, Karakis I, Amitay M, Anis E, Singer S, et al. Prediction of shigellosis outcomes in Israel using machine learning classifiers. Epidemiol Infect. 2018;146(11):1445–51. 10.1017/S0950268818001498 PMC9133678 29880081

[81] Tran H, Friendship R, Poljak Z. Classification of group A rotavirus VP7 and VP4 genotypes using random forest. Front Genet. 2023;14:1029185. 10.3389/fgene.2023.1029185 PMC10267748 37323680

[82] Yehuala TZ, Derseh NM, Tewelgne MF, Wubante SM. Exploring machine learning algorithms to predict diarrhea disease and identify its determinants among under-five years children in East Africa. J Epidemiol Glob Health. 2024;14(3):1089–99. 10.1007/s44197-024-00259-9 PMC11442874 39073532

[83] Liang D, Wang L, Liu S, Li S, Zhou X, Xiao Y, et al. Global incidence of diarrheal diseases-an update using an interpretable predictive model based on XGBoost and SHAP: a systematic analysis. Nutrients. 2024;16(18):3217. 10.3390/nu16183217 PMC11434730 39339819

[84] Kassaw AK, Alebachew Muluneh A, Assefa EM, Yimer A. Predictive modeling and socioeconomic determinants of diarrhea in children under five in the Amhara region. Ethiopia Front Public Health. 2024;12:1366496. 10.3389/fpubh.2024.1366496 PMC11327862 39157521

[85] Zemariam AB, Abey W, Kassaw AK, Yimer A. Comparative analysis of machine learning algorithms for predicting diarrhea among under-five children in Ethiopia: evidence from 2016 EDHS. Health Informatics J. 2024;30(3):14604582241285768. 10.1177/14604582241285769 39270135

[86] Ogwel B, Mzazi VH, Awuor AO, Okonji C, Anyango RO, Oreso C, et al. Derivation and validation of a clinical predictive model for longer duration diarrhea among pediatric patients in Kenya using machine learning algorithms. BMC Med Inform Decis Mak. 2025;25(1):28. 10.1186/s12911-025-02855-6 PMC11737202 39815316

[87] Jin L, Huang Y, Xi J, Zhan L, Jin H, Chen Y, et al. Interpretable machine learning-derived nomogram model for early detection of persistent diarrhea in Salmonella typhimurium enteritis: a propensity score matching based case-control study. BMC Infect Dis. 2025;25(1):199. 10.1186/s12879-025-10587-1 PMC11812143 39930383

[88] Pranesh P, Singh CD, Sivanandam A, Muthusamy R, Sharma S, Alqahtani T, et al. Predictive analysis of pediatric gastroenteritis risk factors and seasonal variations using VGG Dense HybridNetClassifier a novel deep learning approach. Sci Rep. 2025;15(1):23912. 10.1038/s41598-025-08718-4 PMC12227674 40615459

[89] Wynants L, Van Calster B, Collins GS, Riley RD, Heinze G, Schuit E, et al. Prediction models for diagnosis and prognosis of COVID-19: systematic review and critical appraisal. BMJ. 2020;369:m1328. 10.1136/bmj.m1328. PMC7222643 32265220

[90] Collins GS, Dhiman P, Andaur Navarro CL, Ma J, Hooft L, Reitsma JB, et al. Protocol for development of a reporting guideline (TRIPOD-AI) and risk of bias tool (PROBAST-AI) for diagnostic and prognostic prediction model studies based on artificial intelligence. BMJ Open. 2021;11(7):e048008. 10.1136/bmjopen-2020-048008 PMC8273461 34244270

[91] Kotloff KL, Blackwelder WC, Nasrin D, Nataro JP, Farag TH, Van Eijk A, et al. The Global Enteric Multicenter Study (GEMS) of diarrheal disease in infants and young children in developing countries: epidemiologic and clinical methods of the case/control study. Clin Infect Dis. 2012;55(suppl 4):S232–45. 10.1093/cid/cis753 PMC3502307 23169936

[92] MAL-ED Network Investigators. The MAL-ED study: a multinational and multidisciplinary approach to understand the relationship between enteric pathogens, malnutrition, gut physiology, physical growth, cognitive development, and immune responses in infants and children up to 2 years of age in resource-poor environments. Clin Infect Dis. 2014;59(suppl 4):S193–206. 10.1093/cid/ciu653 25305287

[93] Rajkomar A, Dean J, Kohane I. Machine learning in medicine. N Engl J Med. 2019;380(14):1347–58. 10.1056/NEJMra1814259 30943338

[94] Rudin C. Stop explaining black box machine learning models for high stakes decisions and use interpretable models instead. Nat Mach Intell. 2019;1(5):206–15. 10.1038/s42256-019-0048-x PMC9122117 35603010

[95] Shadmi E, Chen Y, Dourado I, Faran-Perach I, Furler J, Hangoma P, et al. Health equity and COVID-19: global perspectives. Int J Equity Health. 2020;19(1):104. 10.1186/s12939-020-01218-z PMC7316580 32586388

[96] Pisani E, Aaby P, Breugelmans JG, Carr D, Groves T, Helinski M, et al. Beyond open data: realising the health benefits of sharing data. BMJ. 2016;355:i5295. 10.1136/bmj.i5295. PMC6616027 27758792

