# Supplementary material for: Predictive modelling approaches for childhood diarrhoeal disease: a systematic review (2000–2025)
Source: Infect Dis Poverty. 2026 Nov 3;15:112. doi: 10.1186/s40249-026-01508-8 (PMC13729606; doi:10.1186/s40249-026-01508-8)
Supplement: Supplementary file 2 [file 40249_2026_1508_MOESM3_ESM.pdf]

Full electronic search strategies

PubMed/MEDLINE

Search strategy:
("child, preschool"[MeSH Terms] OR "infant"[MeSH Terms] OR "infant*"[Title/Abstract] OR "under-5"[Title/Abstract]
OR "under five"[Title/Abstract] OR "toddler*"[Title/Abstract] OR "neonat*"[Title/Abstract]
OR "newborn*"[Title/Abstract])
AND
("diarrhea"[MeSH Terms] OR "gastroenteritis"[MeSH Terms] OR "diarrhea"[Title/Abstract]
OR "diarrhoea"[Title/Abstract] OR "gastroenteritis"[Title/Abstract]
OR "acute diarrhea"[Title/Abstract] OR "dysentery"[Title/Abstract])
AND
("predictive model*"[Title/Abstract] OR "prediction model*"[Title/Abstract]
OR "forecasting model*"[Title/Abstract] OR "forecast*"[Title/Abstract]
OR "machine learning"[Title/Abstract] OR "artificial intelligence"[Title/Abstract]
OR "deep learning"[Title/Abstract] OR "neural network*"[Title/Abstract]
OR "random forest"[Title/Abstract] OR "support vector"[Title/Abstract]
OR "decision tree*"[Title/Abstract] OR "regression model*"[Title/Abstract]
OR "statistical model*"[Title/Abstract] OR "time series"[Title/Abstract]
OR "ARIMA"[Title/Abstract] OR "autoregressive"[Title/Abstract]
OR "XGBoost"[Title/Abstract] OR "gradient boosting"[Title/Abstract]
OR "ensemble"[Title/Abstract] OR "classification"[Title/Abstract]
OR "clustering"[Title/Abstract] OR "LSTM"[Title/Abstract]
OR "convolutional neural"[Title/Abstract] OR "CNN"[Title/Abstract]
OR "GRU"[Title/Abstract] OR "recurrent neural"[Title/Abstract]
OR "risk prediction"[Title/Abstract] OR "risk model*"[Title/Abstract]
OR "early warning"[Title/Abstract] OR "surveillance"[Title/Abstract]
OR "algorithm*"[Title/Abstract] OR "data mining"[Title/Abstract])
AND ("2000/01/01"[Date - Publication] : "2025/9/18"[Date - Publication])
AND English[Language]
NOT (Editorial[pt] OR Letter[pt] OR Comment[pt] OR "Published Erratum"[pt] OR Congress[pt])

Scopus

Search strategy:
TITLE-ABS-KEY(infant* OR "under-5" OR "under five" OR toddler* OR neonat* OR newborn* OR preschool OR "pre-school")
AND TITLE-ABS-KEY(diarrhea OR diarrhoea OR gastroenteritis OR "acute diarrhea" OR "acute diarrhoea" OR dysentery)
AND TITLE-ABS-KEY("predictive model*" OR "prediction model*" OR "forecasting model*"

OR forecast*
OR "machine learning" OR "artificial intelligence" OR "deep learning" OR "neural network*"
OR "random forest" OR "support vector" OR "decision tree*" OR "regression model*"
OR "statistical model*" OR "time series" OR ARIMA OR autoregressive OR XGBoost
OR "gradient boosting" OR ensemble OR classification OR clustering OR LSTM
OR "convolutional neural" OR CNN OR GRU OR "recurrent neural"
OR "risk prediction" OR "risk model*" OR "early warning" OR surveillance
OR algorithm* OR "data mining")

Filters applied:
Publication years: 2000–2025
Language: English
Document type: Article, Review

Web of Science Core Collection

Search strategy:
TS=(
(infant* OR "under-5" OR "under five" OR toddler* OR neonat* OR newborn* OR preschool OR "pre-school")
AND
(diarrhea OR diarrhoea OR gastroenteritis OR "acute diarrhea" OR "acute diarrhoea" OR dysentery)
AND
("predictive model*" OR "prediction model*" OR "forecasting model*" OR forecast*
OR "machine learning" OR "artificial intelligence" OR "deep learning"
OR "neural network*" OR "random forest" OR "support vector"
OR "decision tree*" OR "regression model*" OR "statistical model*"
OR "time series" OR ARIMA OR autoregressive OR XGBoost
OR "gradient boosting" OR ensemble OR classification OR clustering
OR LSTM OR "convolutional neural" OR CNN OR GRU OR "recurrent neural"
OR "risk prediction" OR "risk model*" OR "early warning"
OR surveillance OR algorithm* OR "data mining")
)

Filters applied:
Timespan: 2000–2025
Language: English
Document type: Article, Review

Embase (via Ovid)

Search strategy:
(infant/ or preschool child/ or neonate/ or newborn/
or (infant* or "under 5" or "under-5" or toddler* or neonat*
or newborn* or preschool or "pre-school").ti,ab.)
and
(diarrhea/ or gastroenteritis/
or (diarrhea or diarrhoea or gastroenteritis
or "acute diarrhea" or "acute diarrhoea" or dysentery).ti,ab.)
and
(predictive model/ or machine learning/ or artificial intelligence/
or deep learning/ or time series analysis/
or ("predictive model*" or "prediction model*" or "forecasting model*"
or forecast* or "machine learning" or "artificial intelligence"
or "deep learning" or "neural network*" or "random forest"
or "support vector" or "decision tree*" or "regression model*"
or "statistical model*" or "time series" or ARIMA or autoregressive
or XGBoost or "gradient boosting" or ensemble or classification
or clustering or LSTM or "convolutional neural" or CNN or GRU
or "recurrent neural" or "risk prediction" or "risk model*"
or "early warning" or surveillance or algorithm* or "data mining").ti,ab.

Limits applied:
Year: 2000–2025
Language: English
Document type: Article, Review

Global Health (via Ovid)

Search strategy:
(infant* OR "under-5" OR "under five" OR toddler* OR neonat*
OR newborn* OR preschool OR "pre-school").ti,ab.
AND
(diarrhea OR diarrhoea OR gastroenteritis
OR "acute diarrhea" OR "acute diarrhoea" OR dysentery).ti,ab.
AND
("predictive model*" OR "prediction model*" OR "forecasting model*"
OR forecast* OR "machine learning" OR "artificial intelligence"
OR "deep learning" OR "neural network*" OR "random forest"
OR "support vector" OR "decision tree*" OR "regression model*"
OR "statistical model*" OR "time series" OR ARIMA OR autoregressive
OR XGBoost OR "gradient boosting" OR ensemble OR classification
OR clustering OR LSTM OR "convolutional neural" OR CNN OR GRU

OR "recurrent neural" OR "risk prediction" OR "risk model*"
OR "early warning" OR surveillance OR algorithm* OR "data mining").ti,ab.

Limits applied:
Year: 2000–2025
Language: English
Document type: Journal article

Grey Literature

Google Scholar:
("child*" OR "infant*" OR "toddler*" OR "neonat*" OR "newborn*"
OR "under-5" OR "under five" OR "preschool" OR "pre-school"
OR "pediatric*" OR "paediatric*")
AND
("diarrhea" OR "diarrhoea" OR "gastroenteritis"
OR "enteric infection*" OR "rotavirus" OR "norovirus")
AND
("predictive model*" OR "machine learning"
OR "artificial intelligence" OR "deep learning"
OR "neural network*" OR "risk prediction")
File type: PDF
Year range: 2000–2025

ProQuest Dissertations & Theses:
TI,AB(infant* OR "under-5" OR "under five" OR toddler* OR neonat*
OR newborn* OR preschool OR "pre-school")
AND
TI,AB(diarrhea OR diarrhoea OR gastroenteritis
OR "acute diarrhea" OR "acute diarrhoea" OR dysentery)
AND
TI,AB("predictive model*" OR "prediction model*"
OR "forecasting model*" OR forecast* OR "machine learning"
OR "artificial intelligence" OR "deep learning"
OR "neural network*" OR "random forest" OR "support vector"
OR "decision tree*" OR "regression model*"
OR "statistical model*" OR "time series" OR ARIMA
OR autoregressive OR XGBoost OR "gradient boosting"
OR ensemble OR classification OR clustering OR LSTM
OR "convolutional neural" OR CNN OR GRU
OR "recurrent neural" OR "risk prediction"
OR "risk model*" OR "early warning"
OR surveillance OR algorithm* OR "data mining")

Limits applied:
Year: 2000–2025
Language: English
